# Supplementary material for: The Reliability and Medical Students’ Appreciation of Certainty-Based Marking
Source: Int J Environ Res Public Health. 2022 Feb 2;19(3):1706. doi: 10.3390/ijerph19031706 (PMC8834968; doi:10.3390/ijerph19031706)
Supplement: Supplementary file 1 [file ijerph-19-01706-s001.zip › ijerph-1532426-supplementary.pdf]

Table S1: Translated VARK questionnaire (in Slovene). For the purpose of this paper, we have included the learner type category to which each of the available answer corresponds. These categories are designated as (V), (A), (R), (K) and are written to the right of each answer. These categories were not written in the questionnaire in the study. (V) - visual, (A) - auditory, (R) - read/write and (K) - kinesthetic.

Vprašalnik o učnih stilih

1. Spletna stran ima video, ki vam prikazuje, kako boste izdelali poseben graf. Na videu govori oseba, video vsebuje še nekaj seznamov in besedila, ki kažejo, kaj morate storiti in še nekaj diagramov. Najbolj bi si zapomnili in se naučili zaradi:
    A. ogleda diagramov (V)
    B. poslušanja (A)
    C. branja besed (R)
    D. ogleda, kaj se v videu dogaja (K)
2. Želite se naučiti kako deluje računalnik. Vi bi:
    A. prebrali pisna navodila, ki so priložena programu (R)
    B. govorili z ljudmi, ki program poznajo (A)
    C. uporabili gumbe ali tipkovnico (K)
    D. sledili diagramom v knjigi, ki je bila priložena (V)
3. Želite najti trgovino, ki vam jo je priporočil prijatelj. Vi bi:
    A. napisali navodila za pot (R)
    B. poiskali, če je v bližini kakšna trgovina, ki jo že poznate (K)
    C. uporabili zemljevid (V)
    D. vprašali prijatelja, da vam pove navodila (A)
4. Rad imam spletne strani, ki imajo:
    A. polja, na katera lahko klikam, jih premikam ali poizkušam(K)
    B. zanimiv design in vizualne značilnosti (V)
    C. zanimive pisne opise, sezname in razlage (R)
    D. avdio kanale, kjer lahko poslušam glasbo, radio programe in intervjuje (A)
5. Kupujete stanovanje ali hišo. Pred ogledom bi si želeli dobiti:
    A. načrt prostorov (V)
    B. natisnjen seznam sob in notranje opreme (R)
    C. video ogled stanovanja ali hiše (K)
    D. pogovor z lastnikom (A)
6. Na kakšen način bi se najraje odločili kako varčevati svoj denar?
    A. za vsak način bi si izračunal koliko bi privarčeval (K)
    B. ogledal bi si grafe, ki kažejo spreminjanje vrednosti (V)
    C. pogovoril bi se s finančnim strokovnjakom (A)

D. prebral bi si brošure (R)

7. Kako se najraje učite?
   A. berem knjige, članke in izročke (R)
   B. pregledam primere uporabe (K)
   C. rad si glasno berem snov (A)
   D. poskušam najti vzorce v snovi (V)
8. Najraje imam učitelja, ki:
   A. izvaja demonstracije, prikazuje modele in kaže praktične primere (K)
   B. omogoča vprašanja in odgovore, govore, skupinske diskusije in vabi gostujoče govorce (A)
   C. uporablja izročke, knjige ali druga pisana gradiva (R)
   D. uporablja diagrame, tabele ali grafe (V)
9. Zaključili ste s tekmovanjem in bi želeli povratno informacijo. Na kakšen način bi jo radi dobili?
   A. z uporabo primerov iz vašega izdelka (K)
   B. z uporabo pisnega opisa vaših rezultatov (A)
   C. od nekoga, ki se z vami o rezultatu pogovori (R)
   D. z uporabo grafov, ki prikazujejo, kaj ste dosegli (V)
10. Imate težavo s srcem. Od zdravnika bi želeli, da:
    A. vam izroči pisno gradivo, da si preberete, kaj je narobe (R)
    B. uporabi plastičen model, da vam pokaže, kaj je narobe (K)
    C. vam opiše, kaj je narobe (A)
    D. vam pokaže diagram, kaj je narobe (V)
11. Želite si izvedeti več o potovanju na katerega greste. Kaj bi storili?
    A. vprašali bi vodiča, s katerim gremo na potovanje (A)
    B. prebrali bi si opis potovanja na spletu (R)
    C. pogledali bi si zemljevid, kjer je označeno kam gremo (V)
    D. pogledali bi si video o tem kaj bomo počeli na potovanju (K)
12. Kaj je za vas pomembno pri izbiri kariere?
    A. dizajn izdelkov, risanje zemljevidov in grafov (V)
    B. dobra uporaba besed v pisni komunikaciji (R)
    C. uporaba znanja v resničnem življenju (K)
    D. komunikacija z drugimi preko diskusije (A)
13. Želite delati na novem projektu. Da ga spoznate si želite:
    A. pogledati grafe, ki kažejo stopnje projekta in porabo denarja (V)
    B. pregledati primere podobnih projektov (K)

C. pogovor z drugimi, ki so že v projektu (A)
D. prebrati zadnje poročilo o projektu (R)

14. Prijateljica vam je prinesla novo družabno igro. Kako bi se jo naučili igrati?
    A. prebral bi navodila (R)
    B. ogledal bi si sheme, ki prikazujejo stopnje igre in poteze (V)
    C. opazoval bi, kako jo igrajo drugi (K)
    D. prosil bi nekoga, da mi razloži igro (A)

15. Želite sestaviti mizo, ki je prišla v delih. Najraje bi uporabili:
    A. priložena navodila, ki opišejo postopek (R)
    B. video, ki kaže kako sestaviti mizo (K)
    C. sheme v navodilih, kjer je prikazano kako se sestavi miza (V)
    D. poklicali bi prijatelja, da vam razloži (A)

16. Želite se naučiti bolje fotografirati. Vi bi:
    A. prebrali knjigo o fotografiranju (R)
    B. uporabili sheme, ki prikazujejo kako pravilno fotografirati (V)
    C. pogledali primere dobrih in slabih fotografij in kako jih popraviti (K)
    D. vprašali bi profesionalnega fotografa (A)
